# Monoaminergic neurotransmitters are bimodal effectors of tau aggregation

**DOI:** 10.1126/sciadv.adr8055

**Published:** 2025-01-31

**Authors:** Xinmin Chang, Amanda M. Tse, Marina Fayzullina, Angela Albanese, Minchan Kim, Conner F. Wang, Zipeng Zheng, Ruchira V. Joshi, Christopher K. Williams, Shino D. Magaki, Harry V. Vinters, Jeremy O. Jones, Ian S. Haworth, Paul M. Seidler

**Affiliations:** ^1^ Department of Pharmacology and Pharmaceutical Sciences, USC Mann School of Pharmacy and Pharmaceutical Sciences, University of Southern California, Los Angeles, CA 90089, USA.; ^2^ Department of Medical Biophysics, Keck School of Medicine, University of Southern California, Los Angeles, CA, USA.; ^3^ Department of Pathology and Laboratory Medicine, David Geffen School of Medicine, University of California, Los Angeles, Los Angeles, CA 90095, USA.; ^4^ Department of Neurology, David Geffen School of Medicine, University of California, Los Angeles, Los Angeles, CA 90095, USA.; ^5^ Brain Research Institute, David Geffen UCLA School of Medicine, Los Angeles, CA 90095, USA.; ^6^ Simulations Plus Inc., 42505 10th Street West, Lancaster, CA 93534-7059, USA.

## Abstract

Neurotransmitters (NTs) mediate trans-synaptic signaling, and disturbances in their levels are linked to aging and brain disorders. Here, we ascribe an additional function for NTs in mediating intracellular protein aggregation by interaction with cytosolic protein fibrils. Cell-based seeding experiments revealed monoaminergic NTs as inhibitors of tau. Seeding is a disease-relevant mechanism involving catalysis by fibrils, leading to the aggregation of proteins in Alzheimer’s disease and other neurodegenerative diseases. Chemotyping small molecules with varied backbone structures revealed determinants of aggregation inhibitors and catalysts. Among those identified were monoaminergic NTs. Dose titrations revealed bimodal effects indicative of fibril disaggregation, with aggregation catalysis occurring at low ratios of NTs and inhibited seeding ensuing at higher concentrations. Bimodal effects by NTs extend from in vitro systems to dopaminergic neurons, suggesting that pharmacotherapies that modify intracellular NT levels could shape the neuronal protein aggregation environment.

## Teaser

Phenolic neurotransmitters influence intracellular protein aggregation through disaggregating effects on fibril-type inclusions.

## INTRODUCTION

Alzheimer’s disease (AD) is a multifactorial disease characterized by accumulations of intraneuronal fibril deposits of tau and extracellular fibril deposits of aggregated Aβ. Antibody therapies targeting Aβ slow the progression of AD dementia (*1*), which emphasizes therapeutic benefits associated with eliminating protein aggregation in AD, although clinical gains remain limited since fibril deposits of aggregated tau continue to be unmanaged. Tau inhibitors, either as a stand-alone therapy or by combination with Aβ monoclonal antibodies, could treat AD more effectively.

Tau aggregation is driven by an autocatalytic seeding cascade (*2*, *3*), which involves templating new fibrils from existing ones. Small-molecule drugs that inhibit tau seeding are highly suitable for central nervous system diseases since they can be tailored to pass the blood-brain barrier (BBB) and cell membranes. Previous research on small-molecule inhibitors identified the binding site and fibril disaggregation mechanism of a polyphenol-type inhibitor, epigallocatechin gallate, for AD tau fibrils (*4*). However, the chemical features of small molecules that define inhibitor potency toward tau remain ill defined. To broadly explore the determinants of small-molecule inhibitors that mediate potency toward tau, we conducted screening using a library of chemically varied phenols.

Screening identified small molecules that were inhibitors and seeding catalysts, several having bimodal concentration–dependent effects on tau. Bimodal effects on seeding are indicative of disaggregant-type inhibitors, which catalyze seeding at low concentrations by increasing seeding-competent nuclei that consist of increased fibril ends. Higher concentrations of the same disaggregants inhibit seeding by eliminating fibril templates and associated seeding activity. Notable among the series of tau effectors we describe here are a handful of monoaminergic neurotransmitters (NTs) such as dopamine (DA), norepinephrine (NE), epinephrine (EP), serotonin, and precursors or metabolites of them.

The bimodal effects of monoaminergic NTs on seeding suggest a pathogenic mechanism by which NT imbalances could foster seeding. In the context of human health, it is possible that the neuronal environment could be primed to either support or combat protein aggregation, depending on the concentrations and compositions of NTs, their precursors, and metabolites with respect to aging and disease. Our data suggest that altered intracellular NT concentrations associated with aging or pharmacotherapy could elicit bimodal concentration–dependent effects, either restoring intracellular NT levels to a homeostatic concentration or disrupting NT levels, potentially promoting protein aggregation. These studies pave the way to understanding the origins of the cellular protein aggregation environment and identifying disease-modifying agents that alter the cytosolic concentrations of monoaminergic NTs.

## RESULTS

### Chemical features of tau effectors found by library screening

**Fig. 1.**
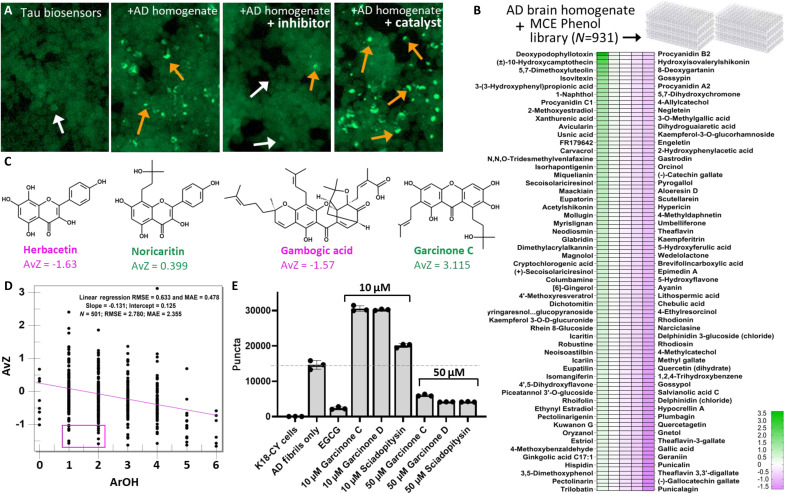
Tau inhibitors identified through phenol library screening. (**A**) Fluorescent micrographs showing representative nonseeded tau biosensor cells without puncta (white arrow), or seeded by transfection with AD brain homogenate, which results in a green punctated appearance (orange arrows). Effects of tau seeding inhibitors and catalysts are shown in representative fluorescent micrographs, as indicated in the image header. (**B**) Heatmap from the MCE phenol library screening with seeding measured from tau biosensor cell assays. Values are expressed as the number of SDs seeding was for a given phenol above (green) or below (magenta) the mean level of seeding from two datasets that comprise biological replicates. Chemicals with seeding at the mean level are colored white in the heatmap. Note: Labels are only present for molecules on the extreme left and right sides of the heatmap. A complete table of molecules and AvZ values can be found in data S1. (**C**) Examples of chemicals and structures of seeding inhibitors and catalysts with related backbones identified by phenol screening, shown for herbacetin versus noricaritin, and gambogic acid versus garcinone C. (**D**) Effect of numbers of ArOHs on seeding. Chemicals with greater numbers of ArOHs exhibit a decreasing trend in AvZ values displaying a negative slope in the line of best fit prepared using ADMET Predictor software. Magenta box shows that a subset of chemicals with as few as one or two ArOHs can strongly inhibit seeding. (**E**) Aggregation catalysts are actually weak tau inhibitors. Garcinone C, garcinone D, and sciadopitysin increase seeding at 10 μM final concentration on cells, but inhibit seeding at 50 μM, which is consistent with results expected for inhibitors acting weakly as fibril disaggregants.

To investigate the chemical characteristics of phenol-type tau inhibitors, we screened the MCE Phenols Library, a collection of 931 phenols that are varied in backbone structures and chemical features. Seeding was measured by transfecting tau biosensor cells with AD brain homogenates following preincubation with library chemicals. Here, biosensors are human embryonic kidney (HEK) 293 cells expressing a yellow fluorescent protein (YFP)–fused aggregation-prone fragment of tau that produces a diffuse green glow (Fig. 1A, white arrows) (*5*). Transfecting tau biosensor cells with AD brain homogenates results in a punctated phenotype, which reflects intracellular accumulations of seeded tau that are templated by tau fibrils from AD brain homogenates (Fig. 1A, orange arrows) (*6*).

To classify the effects phenols with varying structures from the MCE library have on tau seeding, we generated the heatmap in Fig. 1B. Seeding power for a given phenol is represented using SDs from the mean level of seeding, which was determined using two biological replicates, or AvZ. The strength of seeding is proportional to the magnitude of the AvZ. Phenols having positive AvZ values catalyze seeding, whereas those having negative values inhibit seeding. Phenols presented in the heatmap in Fig. 1B are limited to a high-confidence subset from the MCE screen that were reproduced within 1 SD between biological replicates, and/or phenols that were validated in follow-up dose-response experiments. AvZ values for this high-confidence set may be downloaded in data S1, and these data were used for subsequent analysis in this report.

Analysis of over 175 physiochemical descriptors using ADMET Predictor for 548 high-confidence phenols shows that the number of aromatic hydroxyls (ArOH) was the strongest predictor of AvZ, with a negative slope correlating with increasing numbers of ArOH (Fig. 1D). These data indicate that greater numbers of ArOH tend to increase inhibitor potency, although crucially a handful of phenol backbones having strong inhibition and just one to two ArOHs are highlighted in the magenta box in Fig. 1D. Among these, we noted 4-methylcatechol, which is the functional side chain of DA, having an AvZ of −1.25. For the full data series, the range in observed AvZ values spanned from −1.69 to 3.63, indicating that 4-methylcatechol is a relatively strong inhibitor of tau seeding.

The MCE phenol series contains a mixture of inhibitors and seeding catalysts, thereby revealing defining characteristics for each. As seen in the heatmap in Fig. 1B, just over half the phenols (53%) had no effect on seeding. Of the remaining, 14% catalyzed seeding and 33% inhibited seeding based on our analysis applying a cutoff of ±0.5 SDs from AvZ = 0 (i.e., AvZ ≥ 0.5 = catalyst, AvZ ≤ −0.5 = inhibitor, and −0.5 < AvZ < 0.5 = neutral compound, or no significant effect on seeding). A one-way ANOVA comparing inhibitors versus catalysts, inhibitors versus neutrals, and catalysts versus neutrals revealed highly significant differences in average *z* scores between each group, with adjusted *P* < 0.0001 (fig. S1). Both inhibitors and seeding catalysts were found among phenols grouped by chemically similar backbones, indicating that seeding catalysts and inhibitors can be rather similar in structure. Docking inhibitors and catalysts with related backbone structures show that both types are capable of binding the same sites on tau fibrils (table S1). These data suggest that inhibitory and catalytic effects are determined by substituents that affect physicochemical properties of the phenol, rather than deriving from differences in backbone structure or protein binding. Analysis of pairs of phenol inhibitors and catalysts with chemically related backbones shows that inhibitor efficacy generally decreases with aliphatic substituents increasing in number and/or length, as is shown for representative inhibitor (magenta) and catalyst (green) pairs in Fig. 1C.

Aliphatic substituents were chief among the descriptors identified that discern seeding catalysts from inhibitors. Among differing types of aliphatic substituents, aliphatic hydroxyls (AlOH) correlated with increasing AvZ (fig. S2), which is the opposite of the trend that we noted for ArOHs. Further analysis shows that tau inhibitor activity is determined by the electronic and topological environment of the hydroxy substituent. Atom-type electrotopological states (E-states) (*7*, *8*), which combine electronic information including electronegativity and valence state of the hydroxyl and topological information, are shown in fig. S3. The hydroxyl E-state index (SsOH) shows a trend with increasing inhibitor activity (lower AvZ scores) and increasing SsOH, indicating sensitivity of tau to the electronic and topological environment of the hydroxyl. Phenols having intermediate E-state indices of around 40 particularly coincided with negative AvZ scores. These data reveal that the electronic environment of a hydroxyl on a small-molecule ligand is key in determining the catalytic or inhibitory effects that phenols have on tau seeding activity.

### Aggregation catalysts are seeding inhibitors with suboptimal activity

We hypothesized that catalytic effects on seeding that are seen for some phenols in Fig. 1 could be a consequence of ineffective fibril disaggregation, which is expected to enhance puncta by exposing greater concentrations of fibril ends that catalyze seeding. We tested the hypothesis that aggregation catalysts are actually tau inhibitors screened at suboptimal concentrations by selecting a handful of aggregation catalysts from our library to assay in biosensor cells at varied concentrations. As shown in Fig. 1E, garcinone C, garcinone D, and sciadopitysin each catalyzed seeding at 10 μM, but inhibited seeding at 50 μM. These data demonstrate that “weak” tau inhibitors, those having median inhibitory concentrations (IC_50_s) in the high micromolar range, can have bimodal effects on seeding, catalyzing at suboptimal (low) concentrations but inhibiting at high concentrations.

**Fig. 2.**
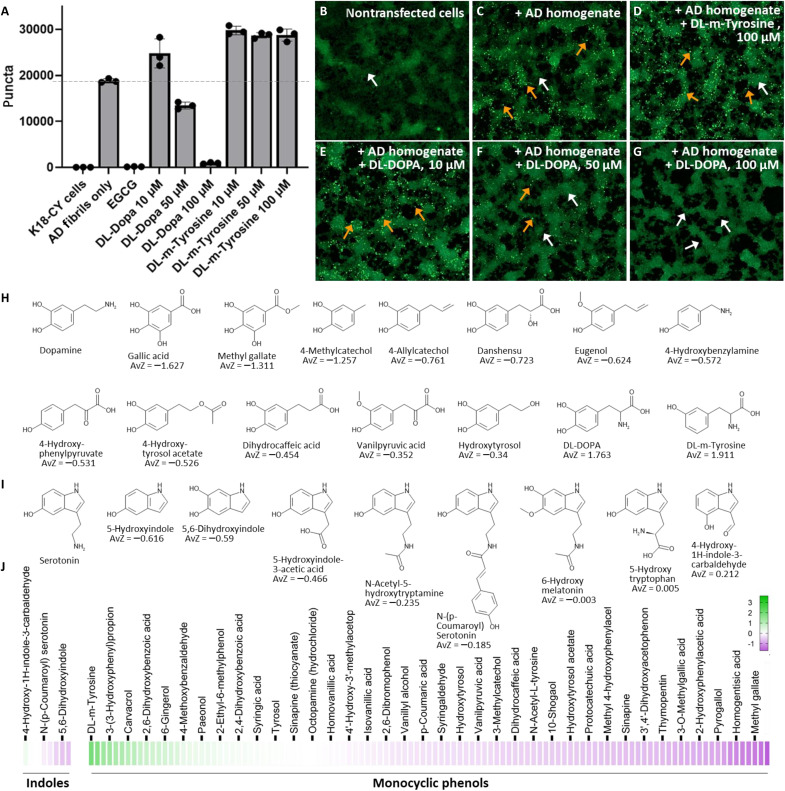
Dose-dependent effects of NT-like backbones from the MCE phenol library series. (**A**) Biosensor seeding by AD brain homogenate pretreated with indicated amounts of dl-dopa or dl-m-Tyr. (**B** to **G**) Example images of cells. Nonseeded cells (white arrow) have no puncta. Transfecting with AD brain homogenate yields subcellular puncta (orange arrows). (**H**) Chemical structures of DA and chemically related analogs identified from the MCE phenol series. AvZ scores are listed for each respective phenol from library screening. (**I**) As in (G), except for analogs of serotonin. (**J**) Heatmap showing range of AvZ scores from the MCE phenol series for indole and monocyclic type tau inhibitors from the MCE library. Generally, both series, which relate to serotonin and DA, contain a preponderance of seeding inhibitors (pink), although phenols having catalytic effects on seeding (green) are also present.

Among the aggregation catalysts uncovered by our library screen was dl-dopa, with an AvZ of 1.76. Similarly, we noted that dl-m-Tyr, a racemic mixture of meta-tyrosine, had catalytic effects on tau seeding. dl-Dopa is the racemic mixture of d- and l-dopa. Tyr is the metabolic precursor to l-dopa. We hypothesized that dl-dopa and dl-m-Tyr could be other weak tau inhibitors with bimodal, concentration-dependent effects on seeding. Therefore, we assayed seeding by tau with dl-dopa and dl-m-Tyr at concentrations ranging from 10 to 100 μM. While dl-m-Tyr catalyzed seeding independently of dose at all concentrations tested (Fig. 2, A to D), dl-dopa flipped from a seeding catalyst at 10 μM seeding to a dose-dependent inhibitor at higher concentrations (Fig. 2, A and E to G).

### Phenolic NTs are tau inhibitors with bimodal effects

Surveying the MCE library identified additional phenols that are similar in structure to NTs. Using a Tanimoto similarity cutoff of 0.65, we identified 120 chemicals with similarity to NTs: 8 relating to the indole of serotonin, 1 relating to the choline backbone of acetylcholine, and 111 relating to the monocyclic phenolic structure of catechols (table S2). The AvZ scores ranged from −0.616 to 0.212 for the indole series and −1.627 to 1.911 for the monocyclic phenol series. The single choline analog in the library, sinapine, had an AvZ of −0.633. Inspecting its chemical structure suggests that addition of one ArOH likely accounts for its observed weakly inhibitory activity toward tau seeding (fig. S4). Heatmaps and representative chemical structures for the indole and monocyclic series are shown in Fig. 2 (H to J) for phenols having the most negative and positive AvZ values. Overall, trends noted in the above are recapitulated in the indole and monocyclic series. That is, highest potency inhibitors have relatively small, nonextended backbones, and AvZ scores generally increase for analogs with aliphatic substituents that increase in length, number, and/or incorporation of AlOHs (fig. S5).

**Fig. 3.**
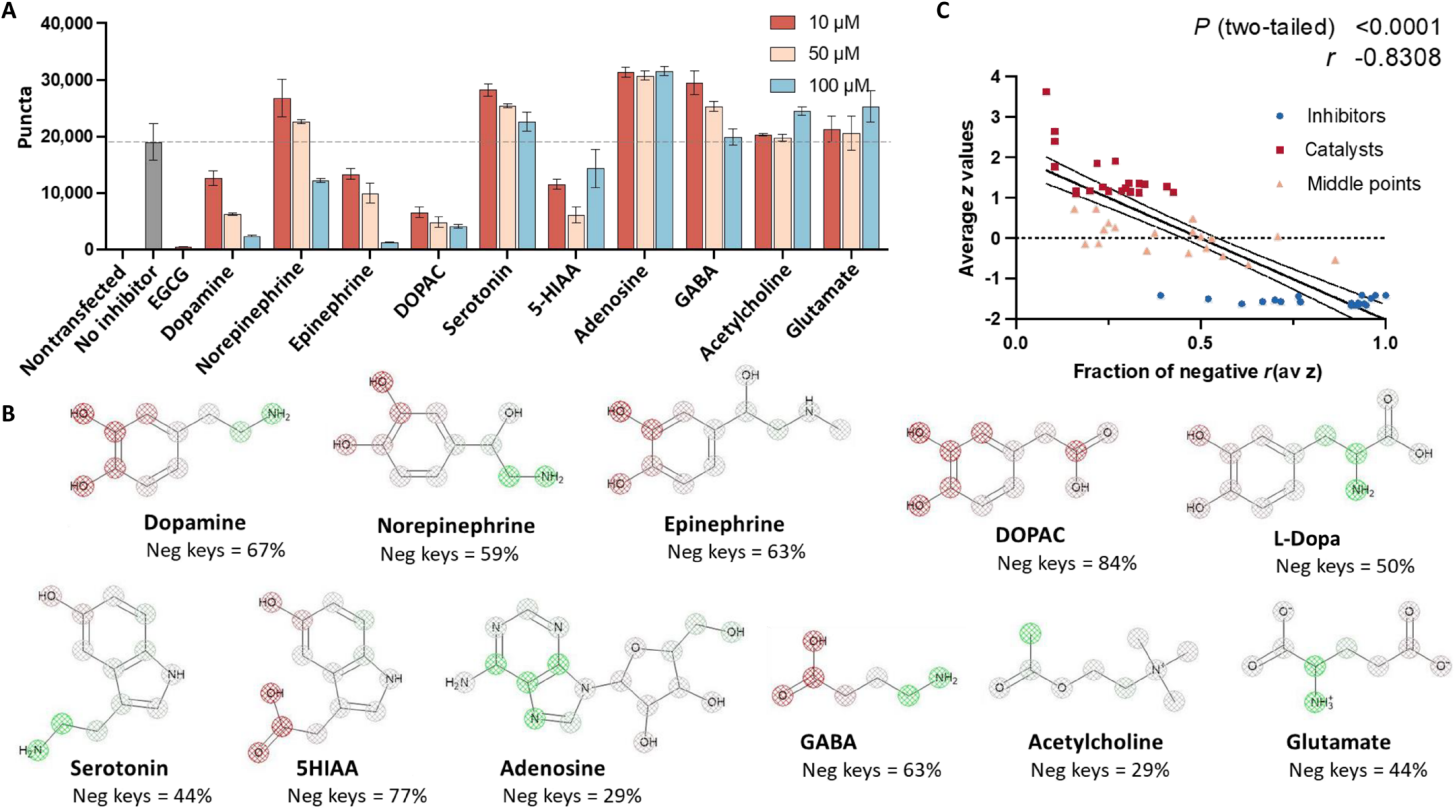
Dose-dependent effects of NTs on tau seeding. (**A**) Biosensor seeding by AD brain homogenate pretreated with indicated amounts of indicated NT. (**B**) SSA for NTs. SSA was conducted using ECFP keys in ADMET Predictor. Atoms depicted with red mesh indicate correlation to negative AvZ values (inhibited seeding). Atoms in green mesh indicate correlation with positive AvZ values (seeding catalysis). Atoms in gray mesh are considered to have negligible effects on seeding. (**C**) Semiquantitative analysis of key negative and positive keys from SSA of select MCE library compounds. Phenols having a higher fraction of negative keys correlate with more negative experimental AvZ scores. Spearman’s rank correlation was computed to assess correlation between the fraction of negative keys and AvZ. There was a negative correlation between the two variables, *r* = −0.8213, *P* < 0.0001.

Since dl-dopa and numerous other monocyclic phenols relating in structure to catecholaminergic NTs were found among the MCE series, we expanded our testing in experiments shown in Fig. 3 to investigate the dose-dependent effects NTs have on tau seeding. As shown in Fig. 3A, DA potently and dose-dependently inhibited tau seeding with effects that are more pronounced than dl-dopa. The differences in activities of DA and dl-dopa are most notable comparing the 10 μM concentration, at which tau seeding is inhibited by DA (Fig. 3A) but catalyzed by dl-dopa (Fig. 2A). NTs from the catecholaminergic series, EP and NE, inhibited seeding with the next greatest efficacy, although effects were weaker than DA (Fig. 3A). The DA metabolite 3,4-dihydroxyphenylacetic acid (DOPAC) also inhibited seeding with effects similar to DA.

Nonphenol NTs, such as serotonin, adenosine, γ-aminobutyric acid (GABA), acetylcholine, and glutamate, exhibited no significant inhibitory effects on tau seeding (Fig. 3A and figs. S6 and S7), although 5-hydroxyindoleacetic acid (5-HIAA), the primary serotonin metabolite excreted in urine, inhibited tau seeding at levels comparable to EP (Fig. 3A). Among the nonphenolic NTs tested, adenosine was the only that dose-dependently catalyzed tau seeding, and this effect was observed exclusively at high concentrations in excess of 10 μM (fig. S7).

### Determinants of inhibitor activity on tau seeding by NTs

Data in Fig. 3A showing relatively weaker inhibitor activity for EP and NE compared with DA agree with observations we made based on the analysis of library compounds from the MCE series, which shows that the presence of AlOHs tends to reduce inhibitor activity. This interpretation is also supported by structure sensitivity analysis (SSA) for DA, NE, and EP that is shown in Fig. 3B. SSA generated using ADMET Predictor applies structural keys to correlate chemical fragments with observed activity. SSA of DA, NE, and EP highlights predicted atomistic contributions toward inhibitor activity and is based on performance data obtained using corresponding keys discovered from the MCE library set with no other training data or input from the NTs shown. SSA identifies the meta- and para-ArOHs as contributing mostly to DA inhibitor activity. Addition of the AlOH is seen to reduce predicted contribution of the para-ArOH in NE. EP is predicted to have greater activity than NE given the terminal methylamino tail, which has a less positive contribution toward the predicted AvZ score than the primary amine tail of NE.

Given the apparent predictive power of SSA to discern inhibitor effects on tau seeding using identifiable structural keys, we tested a semiquantitative method of analyzing effects on seeding using a set of 60 MCE library chemicals to surmise what fraction of composite keys best correlates with inhibitors, catalysts, or phenols that are lacking in any effect. We selected 20 phenols from each category. Structural keys were tallied based on having either a negative or positive contribution to the AvZ, and then the ratio of negatively to positively valued keys was plotted as a function of AvZ score in Fig. 3C. Seeding catalysts uniformly have a composite with less than 45% negative keys, whereas inhibitors are recognized as having greater than 45% of their composite structural keys ascribed with negative AvZ values. Phenols having little-to-no effect on seeding, with AvZ scores near zero identified from the middle point range, varied more widely in their fraction of negative keys, although generally their composition ranged from 20 to 60% negative keys. These data reveal a semiquantitative means of predicting and deciphering the activity of small molecules toward tau seeding on the basis of structural keys. Namely, inhibitors can be recognized as having a majority of negative keys, while catalysts are comprised by structural keys correlating with positively valued AvZ. Small molecules having weak or no effects are comprised by a mixture of structural keys with a slight skew toward positive AvZ values.

Applying the above semiquantitative analysis of structural keys for NTs in Fig. 3B generally predicted experimental measures of seeding. NTs having a high fraction of negative keys were inhibitors of tau seeding. The most effective inhibitor NTs in seeding assays were seen to have a composition of ~60 to 80% negative keys. NTs having less than 45% negative keys had no ability to inhibit tau seeding in seeding assays. GABA, which has a small number of positive keys, was falsely predicted to be a tau seeding inhibitor due to having an inflated fraction of negative keys. Structural keys and their corresponding AvZ values for NTs shown in Fig. 3 are tabulated in data S2.

### NTs alter tau seeding bimodally with differing tau loads

**Fig. 4.**
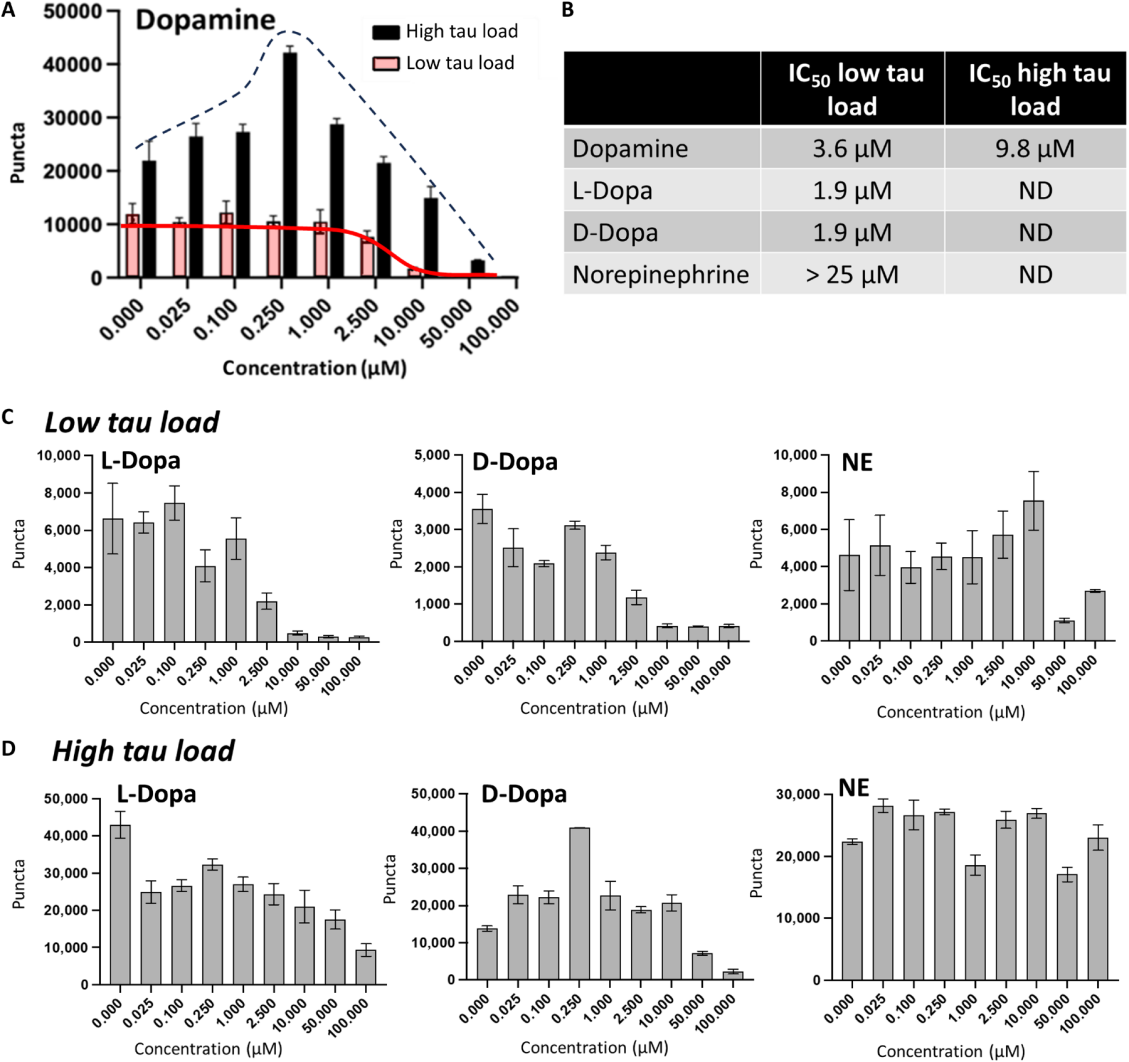
Bimodal effects on seeding are a consequence of NT concentration and tau load. (**A**) Seeding assays conducted using AD brain homogenates diluted to simulate differing tau loads. Treating AD brain homogenate containing a “high tau load” with ptau (1.1 ng/μl) shows a bimodal response to added DA with seeding catalyzed at low DA concentrations and dose-dependently inhibited at high DA concentrations (black bars and interpolated dotted line trace). The same treatment with diluted AD brain homogenate, labeled “low tau load,” containing ptau (0.11 ng/μl) eliminated low-dose catalytic effects of DA on seeding (red bars and interpolated trace). (**B**) IC_50_s computed from curves shown for low and high tau load samples. (**C**) Dose effects of related NTs and metabolites using low tau load AD brain homogenate, as in (A). (**D**) Dose effects of NTs and metabolites from (C) except using high tau load AD brain homogenate.

To scrutinize effects NTs have on tau under a variety of doses and conditions, we tested seeding with varying concentrations and ratios of NTs and tau. DA was tested as an inhibitor at two tau concentrations using AD brain homogenate as a seed with minimal dilution (high tau load), which is the typical assay condition, or AD brain homogenate diluted 10-fold as a proxy to simulate early disease states with low tau fibril loads. Western blotting with AT8 shows that the ptau concentration in the high tau load AD brain homogenate used for seeding was 1.1 ng/μl. Diluting AD brain homogenate to 0.11 ng/μl ptau to obtain a low tau load sample for comparison produced a nonlinear decrease in seeding levels (Fig. 4A, red), which was more potently inhibited by DA with an IC_50_ of 3.6 μM (Fig. 4, A and B). The concentration of DA in the cytosol is in the 1 to 3 μM range (*9*, *10*). These data demonstrate that under conditions with low tau loads, at physiological concentrations, DA inhibits tau seeding in a manner that is absent of catalytic, bimodal effects.

Experiments using high tau load AD brain homogenate as a seed reveal bimodal effects by DA that are apparent in Fig. 4 (A and B). Under conditions with high tau fibril loads, low concentrations of DA in the 0.25 to 2.5 μM range are catalytic toward seeding (Fig. 4A, black dotted trace). Increasing concentrations of DA with the same tau load show inhibited seeding with an IC_50_ of 9.8 μM (Fig. 4, A and B). These data illustrate an important balance involving tau fibril loads and DA by demonstrating bimodal effects on seeding when DA levels are low, tau fibril loads are high, or co-occurrence of the two scenarios.

Dose effects of other catecholaminergic NTs and DA prodrugs were tested using low- and high- tau load AD brain homogenates as a seed (Fig. 4, C and D, respectively). With low tau loads, l- and d-dopa inhibited tau seeding (Fig. 4, B and C) more potently than NE. Contrary to d-dopa, effects of l-dopa lacked catalytic activity toward seeding with high tau loads and low NT concentrations (Fig. 4D). These data agree with MCE library data that showed bimodal effects on seeding by dl-dopa, which is a mixture of the pure enantiomeric forms tested here. Seeding data using enantiomerically pure dopa in Fig. 4D suggest that catalytic effects on seeding at high tau loads derive from the D enantiomer, not the biologically relevant L enantiomer. Weaker responses with a similar overall trend were obtained by varying doses of NE (Fig. 4, C and D). While seeding using low tau load AD brain homogenate was inhibited by high concentrations of NE with an estimated IC_50_ of >25 μM, no inhibition was observed using high tau load AD brain homogenate as a seed.

### DA disaggregates tau fibrils with bimodal effects on seeding in neurons

**Fig. 5.**
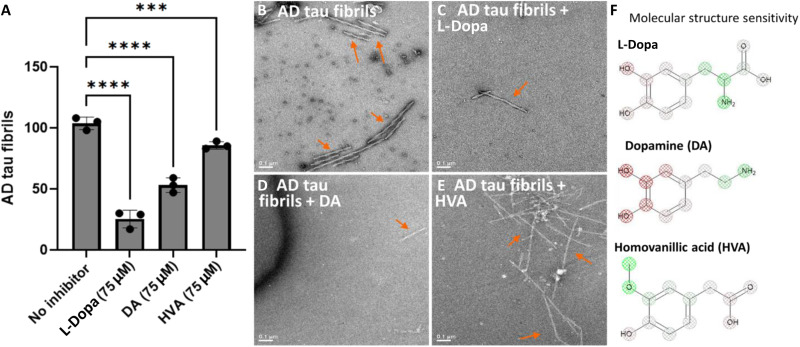
Aromatic hydroxyls mediate fibril disaggregation by dopaminergic molecules. (**A**) Results of qEM showing AD tau fibrils remaining after 24-hour treatment with 75 μM dopaminergic molecules. Fibrils were quantified from three sets of images, with *N* = 32 images per set. Error bars represent SDs from triplicate measures. l-Dopa and DA significantly reduce AD tau fibrils compared to the no inhibitor control, with highly significant *P* values (*P* < 0.0001). HVA led to a significant reduction in fibrils, though to a lesser extent, with *P* = 0.0006. (**B** to **E**) Representative EM images of fibrils without versus with treatment. Arrows point to representative tau fibrils. (**F**) Atomistic contributions toward tau inhibitor activity based on structure sensitivity analysis.

**Fig. 6.**
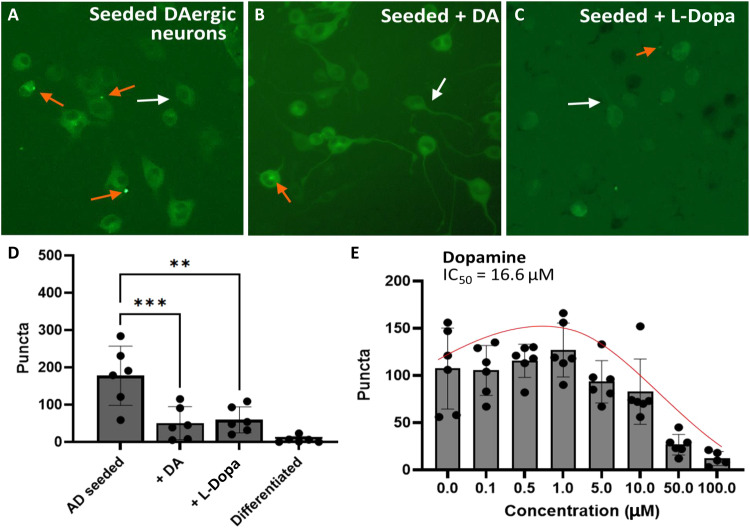
Effects of dopaminergic molecules on seeding in differentiated N2a dopaminergic biosensor cells expressing P301S 4R1N tau fused with YFP. (**A** to **C**) Representative cell images showing seeding, and effects by DA and l-dopa. In all conditions with drug treatment, the final concentration was 50 μM on cells. Examples of cells with puncta are marked by orange arrows, and cells without puncta are marked by white arrows. (**D**) Quantification of puncta in control and seeded cells treated with DA and l-dopa. DA treatment significantly reduces puncta (*P* < 0.001), while l-dopa treatment also results in a statistically significant reduction (*P* < 0.01). (**E**) Dose-dependent effects of pretreatment with DA on tau seeding. Puncta were quantified from two arbitrary images per well, each for three technical replicates. Error bars show SDs.

Bimodal effects on seeding are indicative of fibril disaggregation. Therefore, we tested the bimodal effects of DA in the following ways: measuring in vitro fibril disaggregation by quantitative electron microscopy (qEM) in Fig. 5 and treating seeded dopaminergic neurons with drugs that affect intracellular DA levels in Fig. 6. For qEM, tau fibrils purified from AD brain were preincubated for 24 hours with 75 μM dopaminergic molecules. Fibrils deposited on negatively stained EM grids were counted from *N* = 96 images for each of the dopaminergic molecules tested to obtain the data shown in Fig. 5 (A to E). DA and l-dopa reduced tau fibril counts by about 50 and 75%, respectively, supporting the interpretation that bimodal activity by dopaminergic molecules is attributable to fibril disaggregation. SSA in Fig. 5F highlights prominent atomistic contributions by ArOHs from these dopaminergic molecules, consistent with their predicted roles in fibril disaggregation. By contrast, homovanillic acid (HVA), the primary metabolite of DA, did not appreciably reduce tau fibril load (Fig. 5, A and E), likely due to methylation of one of its ArOHs (Fig. 5F). HVA also did not appreciably inhibit seeding, having an AvZ of −0.054 in tau seeding assays (data S1). These data demonstrate correlated effects linking tau seeding inhibitor activity with in vitro fibril disaggregation.

Next, we tested effects of drugs affecting dopaminergic levels on seeding in dopaminergic neurons. Neuro-2a (N2a) cells stably expressing the aggregation-prone 4R1N tau mutant P301S fused with YFP were cultured with serum reduction followed by addition of dibutyryl cyclic adenosine monophosphate (dbcAMP) to obtain differentiated dopaminergic neurons, as previously described (*11*, *12*). Experiments in Fig. 6 reveal the impact of altering DA levels in tau-expressing dopaminergic neurons by treating cells with DA or the DA prodrug l-dopa. In experiments shown in Fig. 6 (A to D), DA and l-dopa inhibited seeding as judged by reduction in green fluorescence puncta. To further investigate dose effects of DA on seeding, we conducted dose titrations with DA. As shown in Fig. 6E, under conditions using high tau load samples as a seed in dopaminergic neurons, high doses of DA inhibited seeding with an IC_50_ of 16.6 μM. Similar to the effects by lower DA doses seen in HEK293-based biosensor cell assays, lower doses of DA, around 1 μM, exhibited a catalytic effect on seeding. Overall, these data show that the bimodal effects of DA observed in our in vitro experiments are replicated in neuronal cell cultures.

## DISCUSSION

The effects of altered NT concentrations on synaptic signaling in aging, dementia, and neuropsychiatric diseases are manyfold. Our work here homes in on an additional intracellular role for monoaminergic NTs in mediating the aggregation of proteins by effecting structures and seeding capacity of fibril-type inclusions. The structural chemical features of monoaminergic NTs, particularly those from the catecholaminergic series, overlap with the broader features of phenol-type tau inhibitors that we discovered by library screening, and analysis of structural keys gives way to a structure-based understanding of the features that mediate inhibitor potency. Chief among the chemical features of importance is the number of ArOHs, and these studies demonstrate that chemical backbones having as few as one strategically placed ArOH can function as inhibitors of tau seeding. Other noteworthy chemical features ascertained from analysis of keys in data S2 included terminal carboxylic acids, which contributed favorably toward inhibitor activity, and aliphatic side chains, particularly AlOHs, and amino substituents on tail groups, all of which tended to decrease inhibitory activity.

**Fig. 7.**
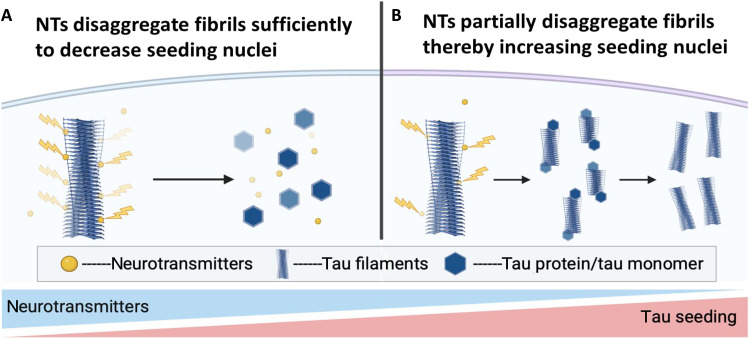
Proposed biological mechanism illustrating how intracellular NTs foster or combat seeding, depending on concentration. (**A**) In cells with sufficient concentrations of monoaminergic NTs and low tau loads, NTs (yellow) could disaggregate tau fibrils as they spontaneously form to keep seeding at bay. (**B**) In cells with low concentrations of monoaminergic NTs and/or high tau loads, monoaminergic NTs are unable to effectively disaggregate fibrils entirely to monomer. Resulting fractured fibrils are seeding-competent nuclei that catalyze tau seeding. Tau monomers are depicted by hexagons. NT concentrations and seeding levels are signified by gradient lines at the bottom. Images were created using Biorender.

Bimodal concentration–dependent effects measured on seeding suggest that catecholaminergic NTs and indoleamine metabolites of serotonin are phenol-type tau inhibitors that function by disaggregating fibrils. As illustrated in the proposed biological model in Fig. 7, NT concentrations in neurons could be an important factor in fibril homeostasis and disease progression. We suggest that in a nondisease state, monoaminergic NTs could help maintain homeostasis by chemically disaggregating fibrils. In disease states, depleted levels of the same monoaminergic NTs could promote cascading protein aggregation through bimodal concentration–dependent effects on seeding due to ineffective fibril disaggregation at low ratios of NTs to tau fibril loads. Thus, it is possible the neuronal environment could be primed to either support or combat protein aggregation depending on the concentration and composition of NTs and levels of their precursors and metabolites.

Is there opportunity for NTs to encounter and bind to tau fibrils inside neurons? Although NTs are destined for compartmentalization inside synaptic vesicles where they reach high concentrations in excess of 1 mM, DA is also present in the cytosol, where it is synthesized, at an estimated concentration of 3 μM (*10*). While there is no evidence suggesting that DA encounters tau fibrils inside synaptic vesicles, it is possible that DA and other monoaminergic NTs could encounter tau fibrils in the cytosol. Tyrosine hydroxylase converts tyrosine to l-dopa, which is subsequently converted to DA by dopa decarboxylase. DA is then transported from the cytosol into synaptic vesicles by VMAT2. There are no known cytoplasmic transporters to facilitate the delivery of cytosolic DA to VMAT2; rather, DA is thought to accrue in low micromolar concentrations in the cytosol until it reaches VMAT2 by diffusion. Its low micromolar cytosolic concentration aligns with the known affinity of VMAT2 for serotonin [5-hydroxytryptamine (5-HT)], another monoaminergic NT, which has a dissociation constant (*K*_d_) of 3.3 μM (*13*). DA exhibits comparable or slightly lower affinity for VMAT2 than 5-HT (*14*). The low micromolar affinities of VMAT2 for monoaminergic NTs suggests that NTs are present in the cytosol at low micromolar concentrations, creating a plausible opportunity for cytosolic monoaminergic NTs, such as DA, to encounter and bind tau fibrils before vesicular uptake.

Although further characterization is needed to understand the effects of intracellular NTs, metabolites, and their dynamically changing concentrations with respect to disease, data suggest that levels of monoaminergic NTs are globally depleted in aging (*15*) and in AD (*16*, *17*), potentially setting the stage for protein aggregation. Studies reporting diminished catecholaminergic NTs—DA, NE, and the precursor l-dopa—link disrupted monoaminergic NT biosynthesis and degradation pathways with NT concentrations and cognitive dysfunction in AD (*17*). Mechanistically, reductions in enzyme levels of phenylethanolamine *N*-methyltransferase (PNMT), which converts NE to EP using DA as a precursor, are linked with AD and neuronal loss in the locus coeruleus (LC) (*18*). Neuronal loss in the LC contributes to alterations in dopaminergic neurons in the hippocampus, which is characterized by depleted hippocampal DA, impaired CA1 synaptic plasticity, neuronal death, and memory deterioration (*16*). Hippocampal DA and NE levels are critically regulated by the LC (*19*), and underscoring the sensitivity of tau aggregation to disrupted DA and NE systems is Braak staging, which demonstrates noradrenergic projection neurons in the LC as the earliest detectable site of tau aggregation pathology (*20*). Clinical imaging data strengthen the neuropathological link between LC integrity, tau aggregation, and memory loss by underscoring a role for tau aggregation in early AD transformations in noradrenergic neurons (*21*).

Collectively, the above previous studies frame AD-related neurodegeneration as originating in the LC, with tau aggregation and coincident depletion of dopaminergic and noradrenergic systems effecting hippocampal plasticity and memory decline. On this basis, it has been suggested that therapeutics targeting tau aggregation and/or levels of catecholaminergic NTs may be conducive to rescuing LC degeneration at the earliest stages of the AD molecular transformation. Our studies add to this body of evidence and further suggest a possible mechanism supporting a link with serotonin and AD (*22*) by showing that the metabolic product of serotonin, 5-HIAA, which is depleted in AD (*23*), is a phenol-type tau inhibitor.

Our study suggests that intracellular concentrations of monoaminergic NTs and their metabolites could be targeted by existing pharmaceuticals for early AD intervention therapy. For example, the DA prodrug l-dopa (levodopa), which is administered orally, is a standard of care treatment for Parkinson’s disease (PD) that is readily accessible. In human studies of AD, levodopa was shown to improve neurophysiological activity, which was suggested by the authors to be a consequence of downstream effects by DA on increasing cholinergic cortical excitability (*24*). Levodopa is transported across the BBB and neuronal cell membranes by LAT1, and is shown to increase intracellular DA concentrations nearly 10-fold, up to 17 μM in neurons treated with levodopa (*25*). The possibility that levodopa itself, or indirectly through increasing DA levels, could inhibit tau aggregation and seeding is plausible but remains unexplored in the clinical setting.

In summary, our experimental work demonstrates that small changes in the concentrations of monoaminergic NTs and/or their related precursors and metabolites can facilitate tau aggregation by partially disaggregating fibrils to enhance seeding. These studies suggest that depleted concentrations of NTs could disturb the cellular environment, creating conditions favorable for tau seeding. Monoaminergic NTs resemble known phenol-type tau inhibitors and fibril disaggregants, providing a mechanistic explanation of how NTs could mediate protein aggregation cascades intracellularly. These studies suggest that the concentrations of monoaminergic NTs are crucial for maintaining protein fidelity in the cellular environment and implicate monoaminergic NT pathways as potential therapeutic target for AD.

## MATERIALS AND METHODS

### Preparation of AD crude brain extracts and purified AD tau fibrils

Human autopsy samples from the University of California, Los Angeles (UCLA) Pathology Department were obtained according to US Department of Health and Human Services regulations from patients consenting to autopsy. Sections of about 250 mg of autopsy brain tissues from human AD patients was cut and then homogenized using a Polytron in 750 μl of sucrose buffer (0.8 M NaCl, 10% sucrose, 10 mM tris-HCl, pH 7.4) supplemented with 1 mM EGTA and 5 mM EDTA. Homogenates, flash-frozen and stored at −80°C, were used directly as tau seeds in biosensor cell assays. To obtain purified tau fibrils from crude brain homogenates, a section of about 2 to 6 g of autopsy brain tissues from human AD patients was cut and then homogenized using a Polytron in 10 ml of sucrose buffer (0.8 M NaCl, 10% sucrose, 10 mM tris-HCl, pH 7.4) supplemented with 1 mM EGTA and 5 mM EDTA. Homogenates were centrifuged at 20,100*g* for 20 min at 4°C, and the supernatants were collected and transferred to airfuge ultra-tubes and spun at 95,000 rpm for 60 min. Ultra-pellet was resuspended in 2.5 ml of sucrose buffer supplemented with 5 mM EDTA and 1 mM EGTA and centrifugated at 20,100*g* for 30 min at 4°C. The supernatant was spun at 95,000 rpm for 60 min at 4°C, and then the pellet containing fibrils was resuspended in 100 μl of 20 mM tris-HCl (pH7.4) and 100 mM NaCl.

### HEK293T tau biosensor seeding assays

Cell seeding assays were conducted using HEK293T cells stably expressing the K18 aggregation-prone tau fragment spanning residues 244 to 372 with two point mutations (P301S and V337M) fused at the C terminus with green fluorescent protein (GFP) (*5*). Cells were cultured in Dulbecco’s modified Eagle’s medium (DMEM) (Life Technologies, catalog no. 11965092) supplemented with 10% (v/v) fetal bovine serum (FBS) (Life Technologies, catalog no. A3160401), 1% penicillin/streptomycin (Life Technologies, catalog no. 15140122), and 1% GlutaMAX (Life Technologies, catalog no. 35050061) in a T25 flask at 37°C in a humidified incubator with 5% CO_2_.

### Phenol screening and *z*-score library transformations

Seeding assays were conducted by transfecting AD brain homogenates in tau biosensor cells. Brain homogenates were preincubated for 16 hours with phenols of interest to yield a 10 μM concentration in cell cultures, except in dose-response assays, which instead used adjustments to achieve the final indicated phenol concentration. Puncta were quantified by high-content imaging and analysis using ImageJ as described previously (*26*) and detailed in the section below. Puncta normalized to cell confluence were taken as the final level of seeding. Library screening was performed with two biological replicates. To normalize the datasets and identify high-confidence hits with reproducible effects on seeding, we conducted *z*-score transformation. Seeding values for each given phenol were subtracted from the average seeding value of the dataset and scaled by dataset’s SD for each of the given biological replicates, as described previously (*27*). Specifically, this was accomplished by calculating the difference between the average number of puncta measured across the entire biological replicate and the number of puncta measured when a given MCE molecule was added. That difference was divided by the SD measured for the same respective dataset. AvZ values were obtained by averaging *z* scores for each given phenol from two biological replicates. Phenols having *z* scores that diverged by greater than 1 SD between biological replicates were excluded from our analysis.

### Image analysis

Images were analyzed using ImageJ software (*28*) to determine numbers of tau puncta in each well. The background fluorescence from unseeded cells was subtracted, and the number of aggregates was counted as peaks with fluorescence above the background using the built-in Particle Analyzer as described previously (*26*). ImageJ scripts can be obtained from Supplementary Text. The results were normalized to the confluence determined by image analysis. For dose titrations, each concentration was repeated in triplicate and the average number of puncta was calculated from triplicate measures. Statistical analyses were performed using GraphPad Prism software.

### Tau biosensor dose titrations

K18-CY cells were plated in a 96-well dish at about 40 to 60% confluence in a volume of 100 μl 24 hours before transfection. Compounds to be tested were preincubated with crude AD brain homogenate [1 μl of homogenates diluted with 19 μl of Opti-MEM (Thermo Fisher Scientific, catalog no. 31985062)] at 4°C overnight before transfecting on cells grown to 60 to 80% confluence. For dose-response assays, nine concentrations were selected, ranging from 0 to 100 μM, in increments of 0, 0.025, 0.01, 0.25, 0.1, 2.5, 10, 50, and 100 μM, and each condition was carried out in triplicate. To enhance seeding, the crude brain homogenates were sonicated in a Qsonica multiple horn water bath for 3 min at 40% power before transfection. For transfection, Lipofectamine 2000 (Thermo Fisher Scientific, catalog no. 11668019) was used according to the manufacturer’s instructions at a 1:20 ratio. Nontransfected cells were the blank group, and only transfected brain homogenate was a positive control for seeding. BioTek Cytation 5 microscopy was used to obtain fluorescent images from seeded 96-well plates. The GFP channel was used, and a 3 × 2 montage mode was set up for the seeded area to capture as much area as possible in each well. The acquired images were analyzed by stitching, preprocessing, and deconvolution for high-content imaging.

### Dopaminergic neuronal biosensor assays

Stable lines expressing tau fused with YFP in mouse N2a were obtained by the following. Cells plated in a 96-well format at confluence were transfected with pEYFP-tau 4R1N-P301S plasmid using Lipofectamine 3000 with P3000 reagent. At 2 days after transfection, geneticin (G418 sulfate) antibiotic was added to the culture medium. The cells were subsequently detached and transferred to a T25 flask for further amplification. YFP-positive cells were sorted via FACSAria Fusion Cell Sorter into low- and high-expressing cells. Before seeding assays, tau-expressing N2a cells were differentiated into dopaminergic neurons as described below.

N2a-expressing tau cells were plated in a 96-well dish at about 30 to 50% confluence in a volume of 100 μl. After 24 hours, cells were differentiated into dopaminergic neurons by FBS reduction to 0.5% and addition of 1 mM dbcAMP sodium salt (Sigma, catalog no. D0627) when cells reached about 50 to 70% confluence. Compounds to be tested were preincubated with crude AD brain homogenate [1 μl of homogenates diluted with 19 μl of Opti-MEM (Thermo Fisher Scientific, catalog no. 31985062)] and incubated at 4°C overnight before transfecting on differentiated cells grown for 2 days after differentiation to develop discernible extended processes. To enhance seeding, the crude brain homogenates were sonicated in a Qsonica multiple horn water bath for 3 min at 40% power before transfection. For transfection, Lipofectamine 2000 (Thermo Fisher Scientific, catalog no. 11668019) was used according to the manufacturer’s instructions at a 1:20 ratio. Nontransfected differentiated cells were the blank group, and only transfected brain homogenate on differentiated cells was a positive control for seeding. Crude AD brain homogenates were preincubated overnight with sufficient drug to achieve a final dose of 50 μM on differentiated cells. All experiments were conducted in triplicate. Fluorescent images were obtained from seeded 96-well plates using the Keyence BZ-X800 microscope employing imaging in the YFP channel with two images captured per replicate at 10× magnification.

### Structure sensitivity analysis

Extended connectivity fingerprint (ECFP) keys were generated using ADMET Predictor, and Key-Attribute correlation was performed using AvZ scores for seeding values measured with phenols from the MCE library set. To quantify the fractional composition of keys for any given phenol, the number of keys with values less than −0.03 was counted as contributing toward a negative AvZ value. Numbers of keys with positive effects on AvZ were similarly tallied for keys with values greater than 0.03. Tallies from each were used to calculate the fraction of negative to positive keys for each given phenol or NT.

### Preparation of negative-stain grids

Purified AD tau fibrils were diluted 1:10 in 1× phosphate-buffered saline (PBS) for negative-stain grids. Diluted samples (6 μl) were pipetted onto the carbon-coated copper grid (TED PELLA, catalog no. 01754-F) and allowed to incubate for 3 min. Excess liquid was removed by blotting the edge of the grid with filter paper. The grid was then quickly treated with 11 μl of negative-stain solution (4% uranyl acetate) for another 2 min before excess stain solution was removed using filter paper. The grid was allowed to air dry for 10 min before being stored in a grid box for further analysis.

### Acquisition qEM images

To obtain qEM images, negative-stain EM grids of each sample were screened at a magnification of ×12,000, and images were collected in 5-μm increments. Fibrils were counted from collections of 100 micrographs for each experimental condition, including purified fibrils with and without 250 μM inhibitors incubated at room temperature for 0 and 24 hours.

### Western blot analysis

Insoluble tau levels in crude brain homogenates were estimated by Western blotting with AT8 antibody. Briefly, 20 μl of crude brain homogenate was heated at 95°C for 20 min in a Bio-Rad polymerase chain reaction (PCR) and then centrifuged at 20,000*g* for 20 min at 4°C. The supernatant was run on a Bolt 4-12% Bis-Tris Plus Gel (invitrogen) in MES SDS Running Buffer (invitrogen). Proteins were transferred to a nitrocellulose membrane (iBlot2 NC Regular Stacks) using the iBlot2 Dry Blotting transfer system (invitrogen) and immunoblotted with AT8 (1:1000, mouse, Thermo Fisher Scientific). A horseradish peroxidase (HRP)–conjugated anti-mouse immunoglobulin G (IgG) secondary antibody (Cell Signaling Technology), together with ECL substrate (Thermo Fisher Scientific), was used for detection. The blot was imaged on an iBright FL 1000 Gel/Cell Imager (Invitrogen). Densitometric quantification of the signals was performed with ImageJ (*28*).
