# Supplementary material for: Monoaminergic neurotransmitters are bimodal effectors of tau aggregation
Source: Sci Adv. 2025 Jan 31;11(5):eadr8055. doi: 10.1126/sciadv.adr8055 (PMC11784839; doi:10.1126/sciadv.adr8055)
Supplement: Supplementary file 1 — Supplementary Text Figs. S1 to S7 Tables S1 and S2 Legends for data S1 and S2 [file sciadv.adr8055_sm.pdf]

Science Advances
AAAS

Supplementary Materials for

Monoaminergic neurotransmitters are bimodal effectors of tau aggregation

Xinmin Chang *et al.*

Corresponding author: Paul M. Seidler, pseidler@usc.edu

Sci. Adv. **11**, eadr8055 (2025)
DOI: 10.1126/sciadv.adr8055

The PDF file includes:

Supplementary Text
Figs. S1 to S7
Tables S1 and S2
Legends for data S1 and S2

Other Supplementary Material for this manuscript includes the following:

Data S1 and S2

SUPPLEMENTARY TEXT

Puncta count script for ImageJ

```
run("Misc...", "divide=Infinity run debug");
run("Subtract Background...", "rolling=0.5 sliding");
run("8-bit");
setAutoThreshold("Default dark");
//run("Threshold...");
setThreshold(14, 255);
run("Close");
run("Analyze Particles...", "  show=Nothing include summarize");
```

Confluence script for ImageJ

```
run("Misc...", "divide=Infinity run debug");
//run("Brightness/Contrast...");
run("Enhance Contrast", "saturated=0.5");
run("Close");
run("8-bit");
setAutoThreshold("Default dark");
setThreshold(8, 255);
//run("Threshold...");
run("Set Measurements...", "area mean area_fraction limit display redirect=None decimal=3");
run("Measure");
```

SUPPLEMENTARY FIGURES

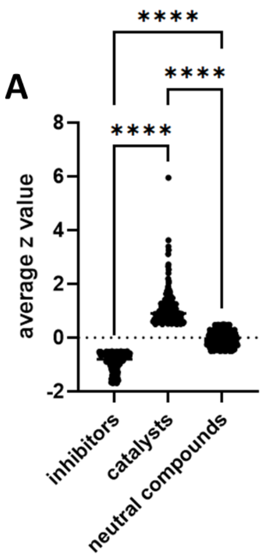

B

Tukey's multiple comparisons test

	Mean Diff.	95.00% CI of diff.
inhibitors vs. catalysts	-1.965	-2.054 to -1.876
inhibitors vs. neutral compounds	-0.8578	-0.9321 to -0.7835
catalysts vs. neutral compounds	1.107	1.031 to 1.184

Figure S1. Statistical significance of differences by class. (A) Polyphenols were categorized into three categories based on AvZ values: $Z \geq 0.5$ = catalyst, $z \leq -0.5$ = inhibitor, $-0.5 < z < 0.5$ = neutral compounds, and analyzed by one-way ANOVA in Prism. Comparisons have an adjusted p-value <0.0001, indicating highly significant differences between each pair of groups. (B) Multiple comparisons test were used to compare significance between different possible groupings. Inhibitors have significantly lower Z-scores compared to both catalysts and neutral compounds, while catalysts exhibit significantly higher Z-scores than neutral compounds. These results suggest that the classification of molecules into inhibitors, catalysts, and neutral compounds is robust and supported by clear statistical distinctions in seeding activity.

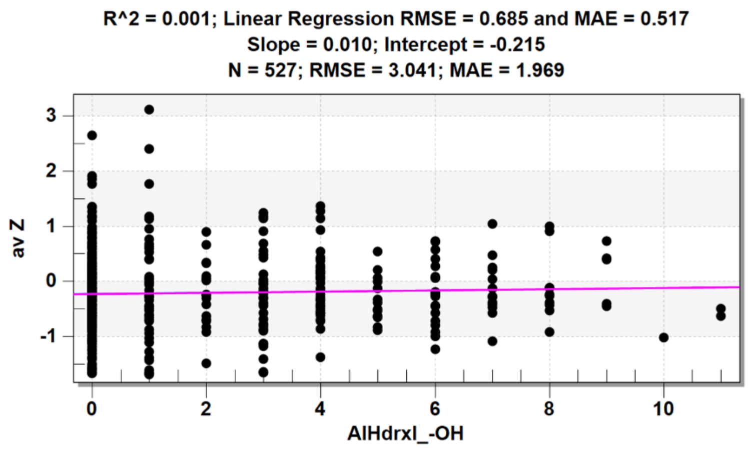

Figure S2. Regression models showing increasing trend between experimental AvZ values and numbers of AlOHs. Analysis was performed using ADMET Predictor®.

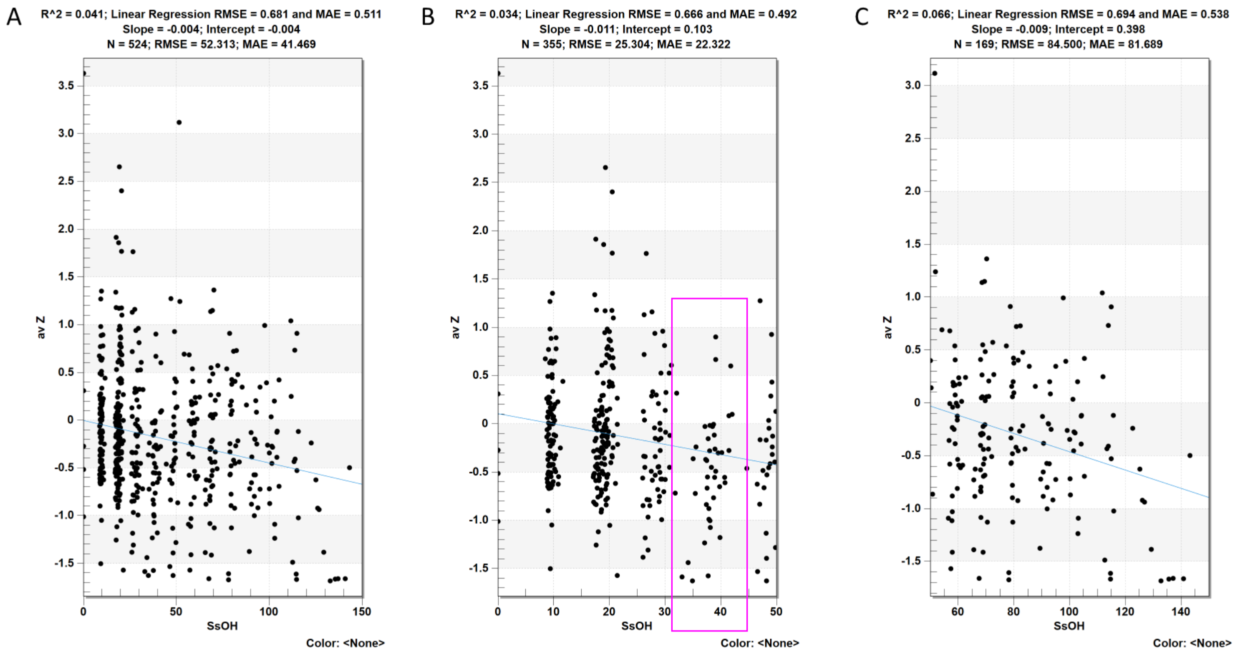

Figure S3. Inhibitor efficacy relates to the electronic and topological environment of the hydroxyl. (A-C) Hydroxyl E-state index (SsOH) is correlated with a negative trending AvZ score indicating tau inhibitor efficacy is sensitive to the electronic and topological environment of the hydroxyl. In A, the entire SsOH range is shown. In B and C, magnified views for low and high range SsOH values are shown, respectively. Phenols having intermediate E-state indices of around 40 in B, marked by the magenta box, particularly coincide with negative AvZ scores. Analysis was performed using ADMET Predictor®.

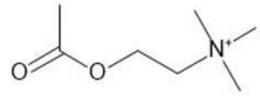

acetylcholine

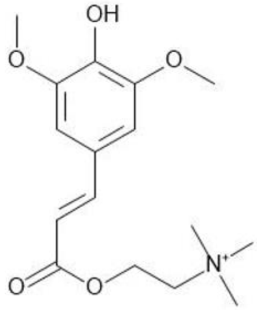

Sinapine

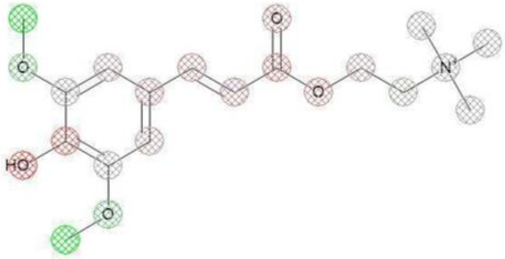

Figure S4. Comparison for acetylcholine and Sinapine, a phenol library chemical that contains a choline moiety. SSA performed using ADMET Predictor® shows the primary inhibitory activity of Sinapine derives from the ArOH, which is absent from acetylcholine.

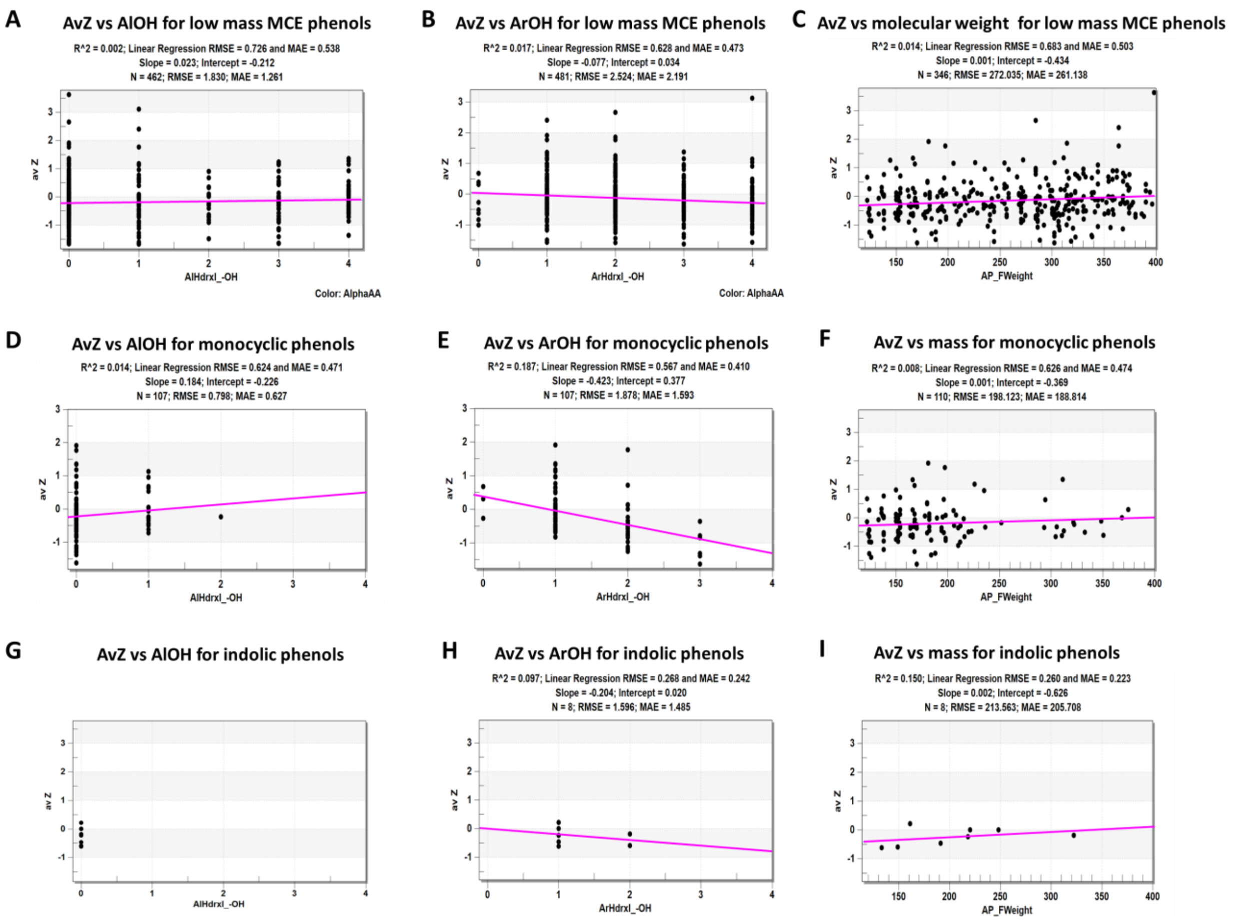

Figure S5. Regression models showing trends between experimental AvZ values and chemical features. Numbers of AlOHs, ArOHs, or molecular mass as a function of AvZ are shown, as indicated. (A-C) MCE chemicals limited in scope to the low mass range (< 400 daltons) matching the monocyclic and indole series presented in D-F and F-I, respectively. (d-f) As in A-C, except for MCE phenols belonging only to the monocyclic series. (G-I) As F-D, except belonging only MCE phenols belonging to the indole series. No variations in AlOH are seen in the indole class in g, and hence a line of best fit could not be obtained. In all other series, a positive increasing trend is seen for AlOHs and molecular mass with AvZ, and a negative trend is seen for increasing numbers of ArOHs with AvZ. Analysis was performed using ADMET Predictor®.

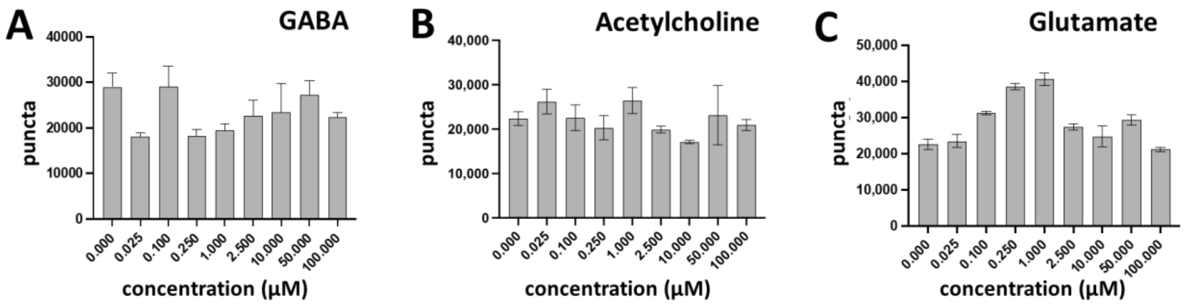

Figure S6. Extended dose series for GABA, acetylcholine, and glutamate. No inhibitory effect on seeding in tau biosensor cell assays is apparent for any of the NT concentrations tested from 25 nM to 100 µM.

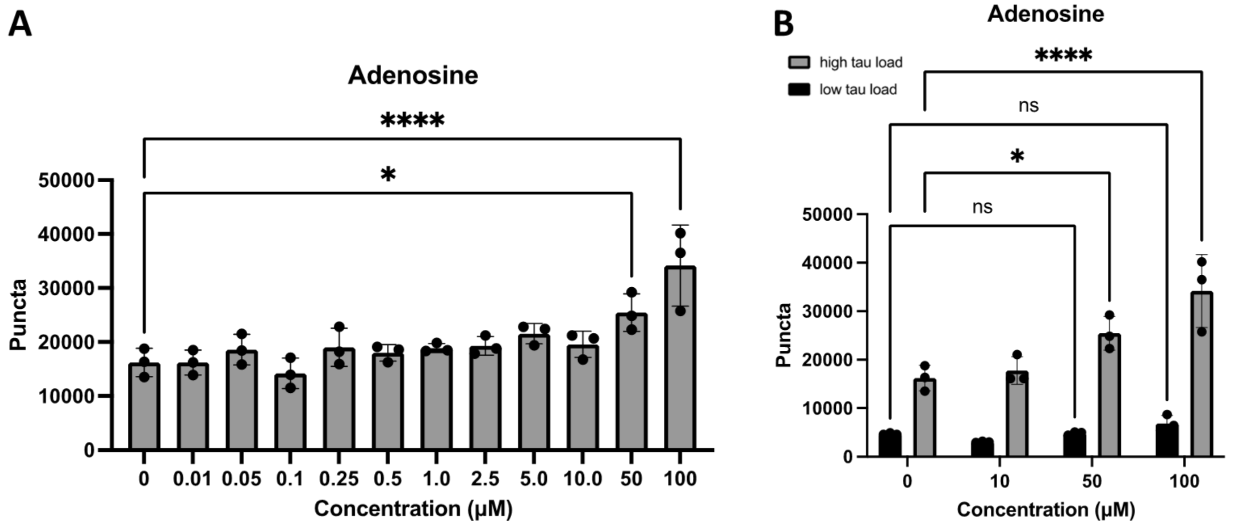

Figure S7. Extended dose series for adenosine. (A) Effects of adenosine on seeding for the extended dose series, shown, for adenosine using high tau load brain homogenate as a seed. Catalytic effects on tau seeding are limited to high concentrations of adenosine, above 10 μM. (B) Effect of tau load on seeding catalyzed by adenosine. Low tau load sample shows less pronounced dose-dependent effects by adenosine on seeding compared with high tau load sample.

SUPPLEMENTARY TABLES

Activity at 10 µM: Inhibitor			Activity at 10 µM: Catalyst		
**Name**	**Chemical Structure**	**Vina Score in EGCG site**	**Name**	**Chemical Structure**	**Vina Score in EGCG site**
Herbacetin	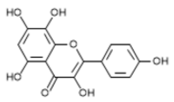	-7.1	Noricaritin	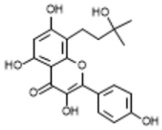	-6.7
Gambogic acid	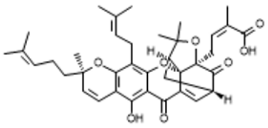	-7.3	Garcinone C	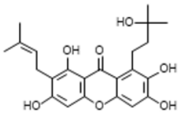	-7.0
Danshensu	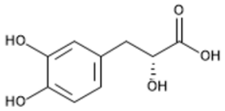	-5.7	3-(3-Hydroxyphenyl) propionic acid	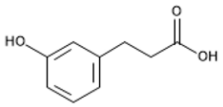	-5.5
Gallic acid	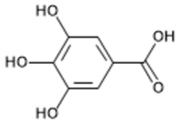	-5.6	2,6-Dihydroxybenzoic acid	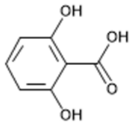	-4.7

Table S1. Docking score for phenol inhibitors and catalysts in the EGCG binding site. Auto blind docking was performed using CB-Dock2[29]. Vina docking scores for chemicals with structures shown are reported in the table. In each case, both inhibitors and catalysts could be docked in the EGCG binding cleft using PDB 7UPG. Inhibitor-catalyst pairs are listed consecutively, and generally lower Vina docking scores are seen for inhibitor phenols relative to a given catalyst with related chemical structure.

MCE Chemical Name	AvZ
***Indoles**:*	
5-Hydroxyindole	-0.616
5,6-Dihydroxyindole	-0.59
5-Hydroxyindole-3-acetic acid	-0.466
N-Acetyl-5-hydroxytryptamine	-0.235
N-(p-Coumaroyl) Serotonin	-0.185
6-Hydroxymelatonin	-0.003
L-5-Hydroxytryptophan	0.005
4-Hydroxy-1H-indole-3-carbaldehyde	0.212
***Cholines**:*	
Sinapine	-0.633
***Monocyclic phenols**:*	
Gallic acid	-1.627
1,2,4-Trihydroxybenzene	-1.385
Methyl gallate	-1.311
4-Methylcatechol	-1.257
Gallic acid (hydrate)	-1.246
Homogentisic acid	-1.185
4-Ethylresorcinol	-1.119
5-Hydroxyferulic acid	-0.968
Pyrogallol	-0.851
Propyl gallate	-0.849
Orcinol	-0.839
2-Hydroxyphenylacetic acid	-0.831
3-Hydroxybenzoic acid	-0.814
Ethyl gallate	-0.79
3-O-Methylgallic acid	-0.786
4-Allylcatechol	-0.761
Danshensu	-0.723
Thymopentin	-0.682
Apocynin	-0.67
Ferulic acid (sodium)	-0.669
3',4'-Dihydroxyacetophenone	-0.667
[8]-Shogaol	-0.665
Guaiacol	-0.653
Sinapine	-0.633
Eugenol	-0.624
10-Gingerol	-0.619
Methyl 4-hydroxyphenylacetate	-0.61
Protocatechualdehyde	-0.588
4-Hydroxybenzylamine	-0.572
Protocatechuic acid	-0.565
4-Hydroxybenzoic acid	-0.554
4-Hydroxyphenylpyruvic acid	-0.531
4-Hydroxytyrosol acetate	-0.526
Isovanillin	-0.519
Salicylic acid	-0.516
10-Shogaol	-0.507
Methyl Salicylate	-0.502
Danshensu (sodium salt)	-0.495
N-Acetyl-L-tyrosine	-0.474

2,5-Dihydroxyacetophenone	-0.464
Caftaric acid	-0.464
Dihydrocaffeic acid	-0.454
Ferulic acid	-0.451
Salicyl alcohol	-0.44
3-Methylcatechol	-0.42
Raspberry ketone	-0.371
2,3,4-Trihydroxybenzoic acid	-0.363
Vanilpyruvic acid	-0.352
Methyl caffeate	-0.349
Nordihydrocapsaicin	-0.342
Hydroxytyrosol	-0.34
Pyrocatechuic acid	-0.338
Isopropyl ferulate	-0.332
Syringaldehyde	-0.318
Capsaicin	-0.318
3-Methoxytyramine	-0.304
p-Coumaric acid	-0.287
Dihydrosinapyl alcohol	-0.284
Isoeugenol acetate	-0.275
Vanillyl alcohol	-0.269
"4-(1,2-Dihydroxyethyl)benzene-1,2-diol"	-0.242
8-Gingerol	-0.236
2,6-Dibromophenol	-0.18
5-Aminosalicylic Acid	-0.176
Homodihydrocapsaicin I	-0.169
Isovanillic acid	-0.151
3-Hydroxybenzaldehyde	-0.137
Anacardic Acid	-0.119
4'-Hydroxy-3'-methylacetophenone	-0.112
Homovanillyl alcohol	-0.068
4-Hydroxybenzyl cyanide	-0.064
Homovanillic acid	-0.054
3-Chloro-L-tyrosine	-0.032
3-Hydroxyphenylacetic acid	-0.027
Octopamine (hydrochloride)	-0.005
2'-Hydroxy-4'-methylacetophenone	-0.002
"2,6-Dimethylhydroquinone"	0.003
Sinapine (thiocyanate)	0.005
3-Hydroxyhippuric acid	0.023
Hydroxyphenyllactic acid	0.039
Tyrosol	0.05
(E)-Methyl 4-coumarate	0.064
Tyramine	0.07
Syringic acid	0.082
Sinapaldehyde	0.096
4?-Hydroxy-2?-methylacetophenone	0.123
2,4-Dihydroxybenzoic acid	0.126
"2,3-Dihydroxy-4-methoxyacetophenone"	0.13
5-Methoxysalicylic acid	0.136
2-Ethyl-6-methylphenol	0.167
DL-Norepinephrine (hydrochloride)	0.246

Mequinol	0.258
Paeonol	0.272
"3,5-Dimethoxyphenol"	0.275
Ginkgolic acid C17:1	0.292
4-Methoxybenzaldehyde	0.308
Propylparaben	0.488
Coniferyl alcohol	0.529
6-Gingerol	0.629
Xanthoxylin	0.65
2-Phenylethanol	0.671
2,6-Dihydroxybenzoic acid	0.715
Creosol	0.768
"N,N,O-Tridesmethylvenlafaxine"	0.955
Carvacrol	0.979
p-Hydroxymandelic acid	1.127
3-Nitro-L-tyrosine	1.177
3-(3-Hydroxyphenyl)propionic acid	1.337
Leonurine	1.35
DL-DOPA	1.763
DL-m-Tyrosine	1.911

Table S2. AvZ scores for tau biosensor cell seeding assays conducted with tau ligands from the HY-L057 Phenols Compound Library from MCE. Molecules exhibiting structural similarity to neurotransmitters (NTs) are categorized by chemotype, with corresponding AvZ values provided to indicate their effects on tau seeding. The categories include chemicals with indole backbones, choline derivatives, and monocyclic phenols.

TITLES AND CAPTIONS FOR THE SUPPLEMENTARY DATA FILES

Data S1. AvZ values for the high-confidence set of MCE phenols. MCE phenols from the HY-L057_Phenols library that reproduced experimentally within 1 standard deviation between two biological replicates, as described in the main text, and/or phenols with activities that were otherwise validated in follow-up dose-response experiments. Corresponding chemical structures for MCE phenols are shown, as are puncta and Z values from each experimental replicate. The table also includes key features such as predicted blood brain barrier permeability and ADMET risk scores.

Data S2. Structural Keys for MCE Phenols with NT-Related Structures. ECFP keys were generated using ADMET Predictor™, and key-attribute correlation was performed based on experimentally determined AvZ values. For each molecule, a Key Name is provided alongside its corresponding molecular fragment, highlighted in red. The contribution of each respective Key to the AvZ value is denoted as r(AvZ). Note: r(AvZ) represents the convolution of the given fragment across the MCE series, as individual fragments are present in multiple molecules, and each molecule itself is composed of multiple Keys.
